# Teleoncology: A Solution for Everyone? A Single-Center Experience with Telemedicine during the Coronavirus Disease 2019 (COVID-19) Pandemic

**DOI:** 10.3390/curroncol29110675

**Published:** 2022-11-11

**Authors:** Paula Ribera, Sandra Soriano, Carla Climent, Laia Vilà, Ismael Macias, Luis Antonio Fernández-Morales, Julia Giner, Enrique Gallardo, Miquel Angel Segui Palmer, Carles Pericay

**Affiliations:** 1 Department of Medical Oncology, Parc Taulí Hospital Universitari, 08208 Sabadell, Spain; 2 Department of Medical Oncology, Hospital Universitari Mútua de Terrassa, 08221 Terrassa, Spain

**Keywords:** telemedicine, teleoncology, COVID-19, pandemic, patient care, oncology care, satisfaction

## Abstract

Since the beginning of the COVID-19 pandemic, the use of telehealth was rapidly implemented without previous evidence. The ONCOTELEMD study aimed to evaluate the opinion of patients attended via telemedicine during this period and to study factors that condition patient preferences on its use. Included patients had a confirmed cancer diagnosis and were contacted by telephone between 13 March and 30 April 2020, in the Medical Oncology Service of Hospital Parc Taulí, Sabadell. A 12-question survey was presented to them between 4 February and 19 April 2021. Statistical analysis was carried out using chi-square and multivariable logistic regression tests. Six hundred forty-six patients were included; 487 responded to the survey. The median age was 68 years (27–90), 55.2% were female. Most patients had a surveillance visit (65.3%) and were diagnosed with colorectal or breast cancer (43% and 26.5%, respectively); 91.8% of patients were satisfied, and 60% would accept the use of telemedicine beyond the pandemic. Patients aged more than 50 years (OR 0.40; 95% CI, 0.19–0.81; *p* = 0.01) and diagnosed with breast cancer (OR 0.45; 95% CI, 0.26–0.69; *p* < 0.001) were less predisposed to adopt telehealth in the future. Patients agreed to be informed via telehealth of scan or lab results (62% and 84%, respectively) but not of new oral or endovenous treatments (52% and 33.5%, respectively). Additionally, 75% of patients had a medium or low-null technologic ability, and 51.3% would only use the telephone or video call to contact health professionals. However, differences were found according to age groups (*p* < 0.0001). In total, patients surveyed were satisfied with telemedicine and believed telehealth could have a role following the COVID-19 pandemic. Moreover, our results remark on the importance of individualizing the use of telehealth, showing relevant data on patient preferences and digital literacy.

## 1. Introduction

The coronavirus disease 2019 (COVID-19) caused by severe acute respiratory syndrome coronavirus-2 (SARS-CoV-2) emerged in Wuhan in late 2019 and became a worldwide pandemic by March 2020 [1].

Since the virus spread, cancer patients were widely accepted as more at-risk due to their underlying disease and more significant immunosuppression [2]. Consequently, the symptoms caused by COVID-19 were more severe, and mortality was also higher compared to the community, reaching a 30% rate according to different series [3,4,5].

To reduce the risk of infection, oncologists were required to readapt cancer care models and evaluate the risks and benefits of outpatient visits in health care centers. Therefore, medical associations like the European Society of Medical Oncology (ESMO) and the American Society of Clinical Oncology (ASCO) recommended the use of telemedicine when it was feasible [6,7].

Telemedicine is defined by the World Health Organization as the delivery of health care services using information and communication technologies to exchange valid information for the diagnosis, treatment, and prevention of disease and injuries, research and evaluation, and the continuing education of health care providers [8].

For cancer care, telemedicine was primarily explored in the 1990s in Australia and the USA to guarantee health care services to rural and remote populations [9]. Afterward, tele-oncology application was not widespread, and its use was restricted to facilitate symptom control, treatment supervision, palliative care, and psychological provision [10,11,12,13,14].

The main drawbacks that have limited the use of telemedicine are the unfamiliarity of patients and caregivers with technology, the impossibility of an in-person physical examination, the compromise of patient–physician interaction and the difficulties for patients with impaired hearing, vision or cognition [15,16,17,18].

Nevertheless, its potential application within cancer care has been acknowledged for a long time. Tele-oncology has some clear benefits for patients and clinicians such as the improvement in punctuality, no need for transportation, lowered waiting time, and reduced time in the hospital environment [19,20,21].

With the arrival of COVID-19, telemedicine was implemented in 76.2% of European centers, and its use increased by 150% compared to 2019 [22,23]. This rapid growth in the provision of telemedicine was performed with limited preceding experience [24].

To support clinicians and health care systems, tele-oncology guidelines were quickly elaborated, recommending telemedicine for routine follow-up/survivorship visits, informing about laboratory and scan results, evaluating oral drug compliance, and managing long-term treatments [6,7,25].

The rapid transition towards telemedicine during the first wave of the COVID-19 pandemic raised concerns about patient acceptance, assessment accuracy, and possible inequalities between patient groups.

Many recent published works report high patient satisfaction with telemedicine during the first pandemic wave, and suggest that telemedicine could have a role in the future [26,27,28,29,30,31,32,33,34,35,36,37,38,39,40]. However, to ensure high-quality care following the pandemic, further investigations are needed to identify patient groups that can benefit more from this new healthcare delivery.

The ONCOTELEMED study was designed during the first wave of the COVID-19 pandemic with three main objectives:

1. Comprehending patient satisfaction with the telehealth visit during this period and finding differences between patient groups.
2. Knowing patient preferences on telemedicine use and identifying the within-group differences.
3. Analyzing the technological skills of our study population and its possible influence on patient preferences towards telemedicine.

With all the information obtained, we would like to answer the question of the optimal patients to receive telehealth care and in which context it should be used.

## 2. Materials and Methods

### 2.1. Study Design and Population

This single-institution descriptive cross-sectional study assessed patients with a reported cancer diagnosis who were contacted via telephone between 13 March and 30 April 2020, in Medical Oncology Service in Hospital Parc Taulí, Sabadell.

Eligible patients were aged 18 years or older. All solid tumours were included. Patients could have an early stage (I–III) or metastatic (IV) disease. Time from diagnosis was irrelevant. Telehealth visits could be for surveillance or treatment follow-up. 

The experience with telemedicine was evaluated with a survey distributed between 4 February and 19 April 2021, ten months after the visit. Temporality was justified given the oversaturation of the Hospital during the first wave of the COVID-19 pandemic. 

We contacted all the included patients by telephone or during an in-person visit (in case the patient had a face-to-face appointment during the survey period). All participants provided their verbal consent before answering the questionnaire. The survey was presented by a trained oncologist who was not involved in patient care. Each survey was filled out only once and anonymously by each participant. 

The study was conducted in accordance with the Declaration of Helsinki, and approved by The Research Ethics Committee of Parc Taulí Hospital on 27 October 2020 with the code number 2020/661.

### 2.2. Survey

A group of different oncologists from our Hospital designed a new patient satisfaction survey. Unfortunately, we could not use a previously validated questionnaire since we could not find an optimal one to answer the primary objectives of our study. Previous works have addressed the satisfaction grade and future perspectives with telemedicine. However, no data existed on patient opinions about what information they should receive during a telemedicine visit. In addition, data on patient abilities with new technology and its influence on telehealth preferences was missing.

The survey contained 12 questions and addressed patient satisfaction with telemedicine, future perspectives, and technological skills. The questionnaire consisted of 7 categorical questions (yes/no/I don’t know), three scale questions, and two multiple-choice questions (Table S1).

### 2.3. Data Collection

Patient demographics and clinical data collection were extracted from the hospital medical records and recorded in a database created ad hoc, including demographics (age, sex), clinical characteristics (ECOG, cancer diagnosis and stage), treatment characteristics (type of treatment and route of administration), and type of visit (surveillance or treatment).

### 2.4. Statistical Analysis

All completed surveys were analyzed. Patient characteristics and survey responses were summarized using descriptive analysis. Mean differences between patient groups were tested for statistical significance using chi-square and logistic regression tests. Significance was considered when *p* < 0.05 (2-sided type I error). Statistical analysis was carried out using the SPSS Package v25.

The analysis of patient satisfaction and the possible differences between study groups was based on Question 2 (*Q2: Did the oncologists’ telephone visit comfort you regarding your disease and its control?*) and performed using a chi-square test. 

To recognize variables associated with the willingness to use telemedicine in the future, we compared dichotomic answers to Question 4 (*Q4: In the future, after the COVID-19 public health crisis, would you accept to switch some in-person visits to virtual?*) using a logistic regression test.

Regarding the influence of patient technological abilities on their preferences towards telemedicine, we considered that age was the only variable of interest. Comparisons, in this case, were also performed using the chi-square test.

## 3. Results

**Figure 1:**
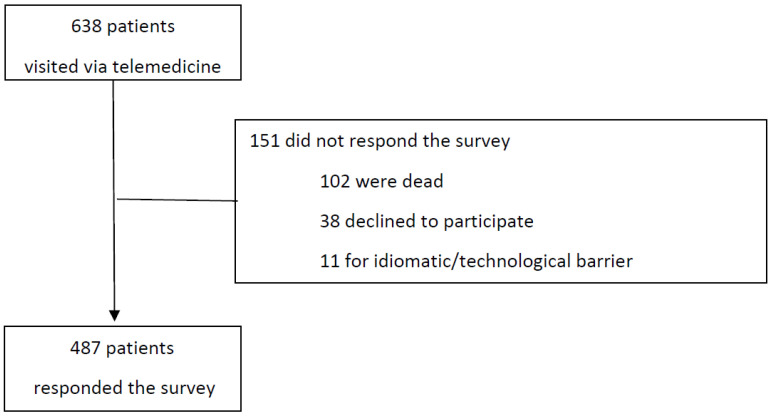
Patients’ flowchart. In total, 638 patients were visited via telemedicine, and 487 answered the survey.

Six hundred thirty-eight patients were contacted via telemedicine between 13 March and 30 April 2020. Four hundred eighty-seven (76.3%) responded to the survey: 57% via telephone and 43% during an in-person visit. The remaining 151 patients did not respond due to death/loss to follow-up (*n* = 102, 16%), refusal to participate (*n* = 38, 6%), or because of an idiomatic/technological barrier (*n* = 11, 1.7%) (Figure 1).

The median age was 68 years old (27–90). Of the respondents, 218 patients (44.8%) were men, and 269 (55.2%) were women. Almost all patients (*n* = 384, 78.9%) were ECOG 0. The most common cancer diagnoses were colorectal (*n* = 210, 43.1%) and breast (*n* = 129, 26.5%). Most patients (*n* = 395, 81%) were diagnosed with an early stage (I–II–III) disease, and only 92 (18.9%) were metastatic (stage IV).

Most patients had a routine surveillance visit (*n* = 318, 65.3%). The remaining 169 (34.7%) had a follow-up visit during active oncologic treatment, which in most cases was hormonotherapy (*n* = 82, 16.8%). Other respondent demographics, cancer, and treatment characteristics are described in Table 1.

### 3.1. Patients’ Satisfaction with the Use of Telemedicine

**Figure 2:**
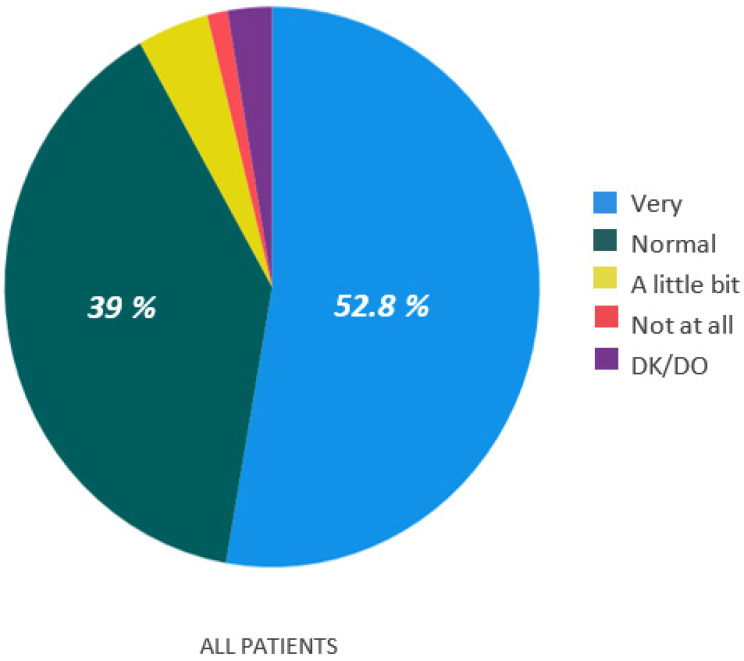
All patients answer to the second question (Q2). *Did the oncologists’ telephone visit comfort you regarding your disease and its control?*

**Figure 3:**
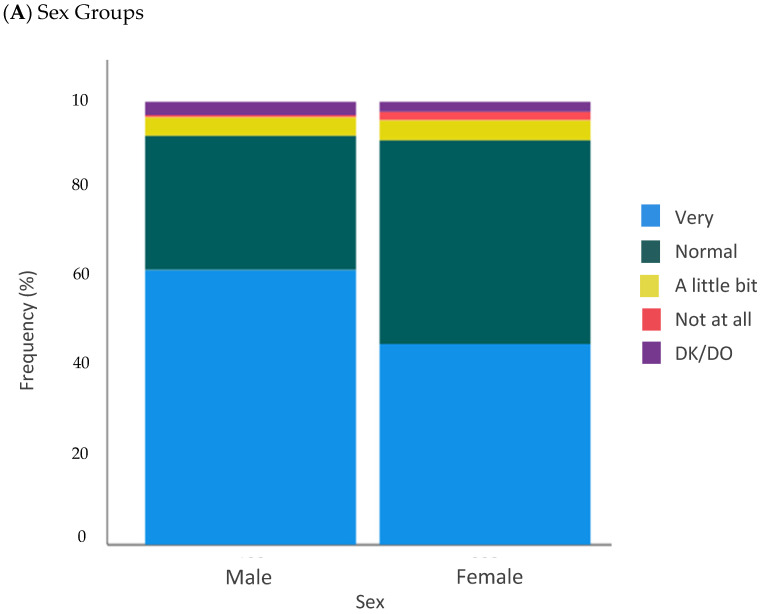

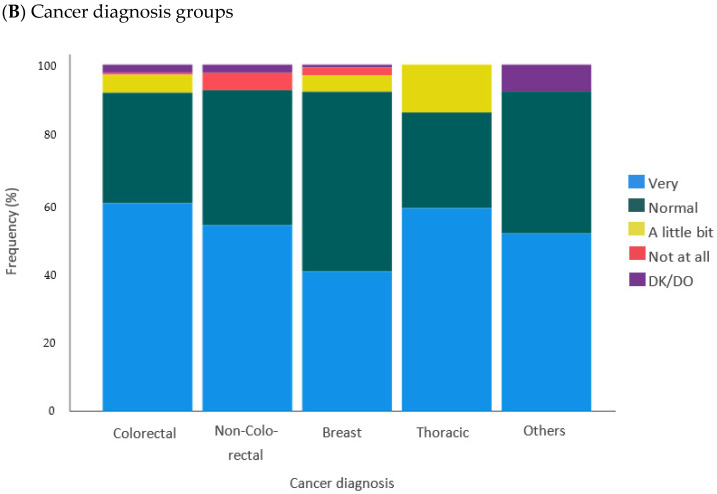
Second question (Q2) regarding sex group (**A**) and cancer diagnosis (**B**). *Q4: Did the oncologists’ telephone visit comfort you regarding your disease and its control?*

For Q2 (*Did the oncologists’ telephone visit comfort you regarding your disease and its control?*), almost all patients responded they felt very (*n* = 257, 52.8%) or normal (*n* = 190, 39%) comforted with the telephone call (Figure 2). The subgroup analysis found significant differences between sex (*p* = 0.003) and cancer type (*p* = 0.001). Our study found that women and breast cancer patients were less comfortable with the telephone visit than men and other cancer types (Figure 3).

No differences were found for the other variables analyzed (age, ECOG, clinical stage, type of visit, oncological treatment, route of administration, and drug type).

### 3.2. Patient Preferences towards Telemedicine and Future Perspectives

When asked for future perspectives (*Q4: In the future, would you accept to switch some in-person to virtual visits?* And *Q5: Do you think telemedicine will play a role in the future?*), 319 patients (65.5%) expressed they would like to switch some in-person to virtual visits, and 308 (63.2%) considered that telemedicine could be part of their health care delivery following the COVID19 pandemic.

**Figure 4:**
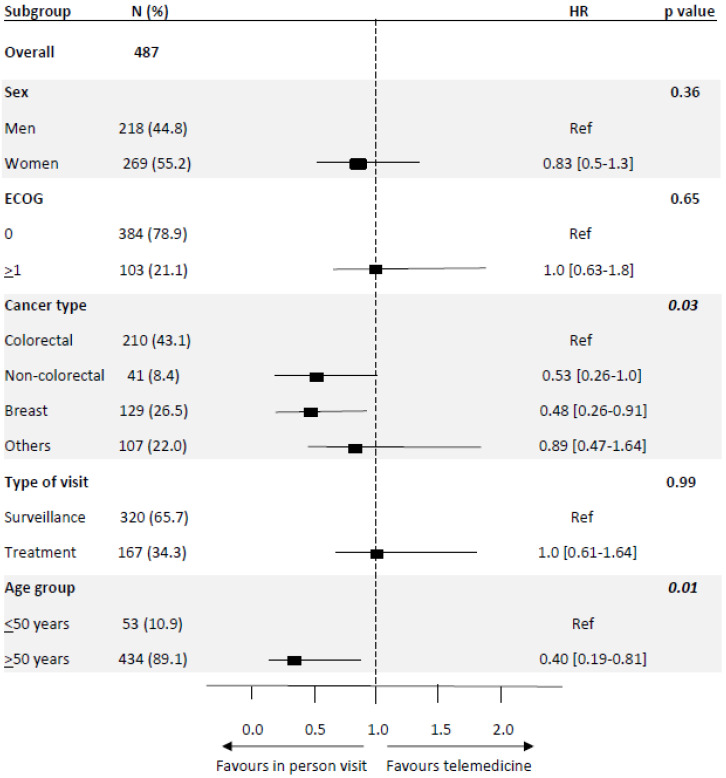
Multivariate logistic regression to assess predictors of telemedicine use willingness in the future. *Q4: In the future, would you accept switching some in-person to virtual visits?*

The univariable analyses for factors associated with willingness to use telemedicine in the future (Q4) showed that women (*p* = 0.02), breast cancer patients (*p* < 0.001), and patients receiving hormonotherapy (*p* = 0.03) were less predisposed to using telehealth. However, in the multivariable analyses to control the expected confounding factors, patients aged more than 50 years (OR 0.40; 95% CI, 0.19–0.81; *p* = 0.01) and diagnosed with breast cancer (OR 0.45; 95% CI, 0.26–0.69; *p* < 0.001) were the less predisposed to changing to telehealth in comparison to younger patients or other cancer types (Figure 4).

Regarding the information given via telemedicine, we analyzed answers to Q6 (*Would you agree to be informed via telemedicine of imaging test results?*) Q7 (*Would you agree to be notified via telemedicine of laboratory test results?*), Q8 (*Would you agree to be informed via telemedicine of an intravenous treatment that you have to start receiving?*) and Q9 (*Would you agree to be informed via telemedicine of an oral treatment that you have to start receiving?*).

**Figure 5:**
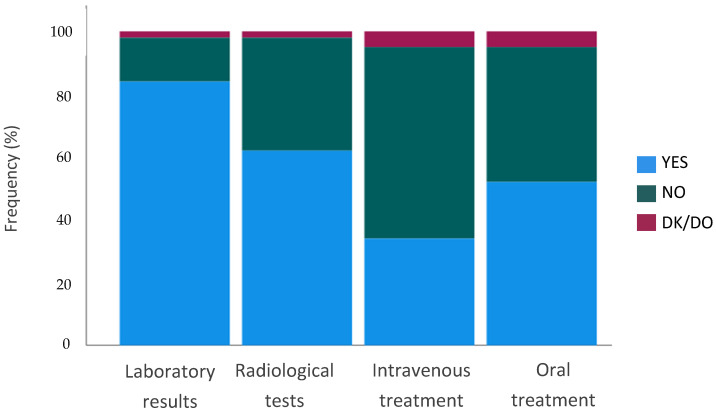
Preferences for the type of information given during a telemedicine visit. All patients. Q6–Q9. *Would you agree to be informed* via *telemedicine of…?*

Most patients would generally accept being informed of radiological tests (*n* = 302, 62%) or laboratory test results (*n* = 410, 84%) during a telehealth visit. However, only 163 patients (33.5%) would like to discuss the indication of intravenous treatments via telemedicine, and 253 (52%) would accept it if treatments were oral (Figure 5). These preferences were similar throughout all groups (sex, age, cancer diagnosis, type of appointment, type of treatment, and route of administration), with statistically non-significant differences in the univariate analysis. 

### 3.3. Patients’ Knowledge of New Technologies

Patients were asked about their abilities with new technologies (*Q11: What is your knowledge of new technologies?*) and the tools they would prefer to use to contact health professionals (*Q12: Which tool would you like to use to reach your oncologist?*).

**Figure 6:**
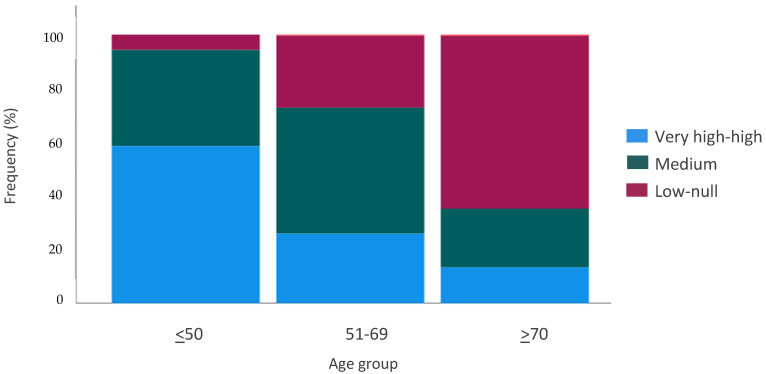
Knowledge of technology. *Q11: What is your knowledge of new technologies?*

For the first question, we observed that the familiarity of the study population with new technologies was poor: 170 patients (34.9%) responded it was medium, and 198 (40.7%) low-null. However, considerable variability throughout the responses was detected according to age groups (*p* = 0.0001). Younger patients (<50 years) were more accustomed to using technological tools (58.5% responded their level was very high-high). In contrast, older patients (>70 years) had more difficulties (64.3% answered their level was low-null) (Figure 6).

**Figure 7:**
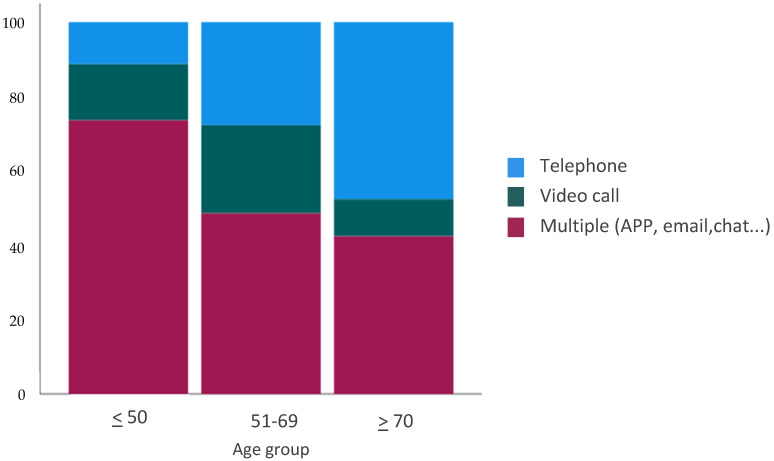
Preference for telemedicine tool. *Q12: Which tool would you like to use to contact your oncologist?*

For Q12, we observed that, in general, patients would prefer to have multiple tools (telephone, video call, chat, APP, email) to contact their doctors (*n* = 237, 48.7%). However, in the subgroup analysis, we found statistically significant differences according to age groups (*p* = 0.0001). For example, most patients older than 70 years would prefer to use only telephone calls (*n* = 100, 47.6%), the majority of patients between 50 and 70 years would prefer only telephone calls (*n* = 62, 27.7%) or video calls (*n* = 53, 23.7%), and patients younger than 50 years would generally use different tools (*n* = 39, 73.6%) (Figure 7).

## 4. Discussion

The COVID-19 pandemic has caused a rapid transition toward telehealth during the last two years. To minimize the risk of infection, telemedicine was implemented for cancer care without previous experience. This fast adoption caused raising concerns about patient satisfaction/preferences and the possible role of tele-oncology in the future.

Numerous recently published works have analyzed patient opinions about the use of telehealth derived from the actual pandemic. In general, telemedicine was well accepted worldwide, reaching satisfaction levels that range from 75 to 92% according to different series [26,27,28,29,30,32,33,34,35,41,42,43]. Our report shows a similar positive telemedicine experience since 91.8% of patients felt comforted by the phone call and could resolve their doubts properly. These results reinforce the strategy adopted in our center during the first pandemic wave when 60% of face-to-face appointments were converted to telehealth visits.

Despite this wide acceptability of telemedicine, a non-negligible percentage of patients (approximately 40%) would not like to switch all in-person visits to teleconsultation and believe telemedicine will not replace existing standards in the future [31]. This information is relevant and suggests telemedicine is not a ‘one size fits all’ option; thus, its implementation requires careful consideration.

There is limited data on the optimal context or patients for which and for whom telemedicine works. In this sense, our work provides relevant and new information by understanding patient preferences and studying the potential inequalities between patient groups with telehealth use.

We report in the present work that willingness to use telemedicine was associated with age or cancer diagnoses: patients with breast cancer and older than age 50 are less predisposed to change.

Regarding breast cancer patients, insufficient evidence existed on telehealth use outcomes before the COVID-19 pandemic. Yan-Ya Chen et al. published a meta-analysis in 2017 showing that telemedicine improved quality of life and reduced psychological distress [44].

With the arrival of COVID-19, considerably more studies addressed breast cancer patients’ telemedicine experience. In general, it was altogether concluded that tele-oncology was effective, safe and that patients were highly satisfied and felt confident with their care [45,46,47,48,49,50,51]. Despite this overall approval, it is important to remark on the matter of the physical examination during a telehealth visit. Lina Cadili et al. [47] exposed in their work that patients with breast cancer felt more anxious due to the impossibility of being examined by the surgeon. The barrier of the physical examination is more limiting for breast cancer care than for other cancer types, given that it is essential to plan the surgery and to detect early recurrences. This limitation could justify that patients with breast cancer were less predisposed to accepting telemedicine as a part of their care delivery.

One of the most critical concerns about the rise of telehealth is equitable access to care, especially for the older adult population. Our results showed that elderly patients were less predisposed to adopting telehealth for cancer care beyond the COVID-19 pandemic. One main reason could be the limited digital access since 64.3% of those older than 70 reported that their familiarity with new technologies was low or null. Moreover, almost half of them would only use the telephone to contact their doctor. Other works have evidenced similar digital health literacy. For example, Lam et al. published a cross-section study in 2020 demonstrating that 38% of older adults were not prepared for video visits and 20% were not prepared for telephone visits, either [52]. Hoogland et al. have also reported that older patients are less likely to own and use smartphones or email addresses [53].

Thus, referring to the selection of optimal patients for telehealth visits and according to our work, patients with breast cancer needing physical examinations and older adults with limited digital access should continue with face-to-face appointments. To increment telehealth use in this scenario, it would be necessary to improve the remote physical exploration with video calls or images and enhance an interdisciplinary team collaboration with family members and caregivers of older adults to guarantee high-quality assistance.

Regarding the type of visit, no differences were found between treatment or surveillance appointments. Both groups were highly satisfied and expressed their willingness to use telemedicine in the future.

The use of telemedicine for surveillance is supported by substantial evidence, especially for managing physical symptoms and psychosocial effects [54]. However, less evidence exists on telehealth use for patients under treatment. Principal limitations are the difficulties in evaluating the performance status, the high acuity of complications, and complex toxicities due to the treatments used.

Concerning the information given via telemedicine visits, most patients expressed their unwillingness to be informed of new intravenous treatments. A non-negligible amount would neither accept to be informed of oral medications nor radiological results.

According to our work, we recommend using telemedicine for short follow-ups during active treatment and some surveillance visits, whereas new treatments and scan results visits require a face-to-face conversation.

Our results need to be interpreted considering the study occurred during the first wave of the COVID-19 pandemic. During that period, telemedicine was rapidly adopted and clinicians and patients did not have adequate knowledge and equipment to obtain the best from telemedicine. This emergency reaction was not planned carefully, so to what extent telemedicine will be a reality following the pandemic is not yet known.

Due to the COVID-19 pandemic and the increasing digitalization of society, telemedicine is likely to keep growing. Prospective data are needed focusing on specific populations and diseases. A key contributor to the applicability of tele-oncology will be digital literacy and appropriate access technology for patients. The principal healthcare system objectives should be the prevention of further inequality and the widening of the digital divide.

This study has some limitations. On the one hand, it is a single-center study, and patients could respond in two different ways: via telephone or during an in-person visit. We also had a small number of patients under treatment (which is not representative of our daily clinical practice), and some tumor types were not represented (gynecological and genitourinary). To conclude, telemedicine was generally accepted during the first wave of the COVID-19 pandemic. However, its use might disadvantage certain patient groups, such as those needing a physical examination, starting new treatments, needing to be informed of scan results, or having poor digital literacy.

## 5. Conclusions

In general, patients in our center were highly satisfied with the use of telemedicine during the COVID-19 pandemic. However, a non-negligible percentage of patients would prefer to continue with conventional care in the future. We found that older age and breast cancer were factors associated with the unwillingness to adopt telemedicine for cancer care. Furthermore, patients preferred to be informed of scan results or new treatment in person.

Although telemedicine will continue to play an essential role in the future, patient preferences and individual situations must be considered. For this reason, to ensure good healthcare, health systems must be encouraged to invest in teaching professionals and patients about the proper use of telehealth.

## Figures and Tables

**Table 1:** Participant demographics, cancer diagnosis, treatment characteristics, and type of visit.

	Respondents *n* = 487 (%)
**Age groups**	
≤50 years	53 (10.9)
51–69 years	224 (46)
≥70 years	210 (43.1)
Gender	
Male	218 (44.8)
Female	269 (55.2)
**ECOG**	
0	384 (78.9)
1	99 (20.3)
≥2	4 (0.8)
**Cancer diagnosis**	
Colorectal	210 (43.1)
GI non-colorectal	41 (8.4)
Thoracic	29 (6)
Breast	129 (26.5)
Others (GIST, melanoma, TNE)	78 (16)
**Clinical stage**	
I	99 (20.3)
II	124 (25.5)
III	172 (35.3)
IV	92 (18.9)
**Type of visit**	
Surveillance	320 (65.7)
Treatment	167 (34.3)
**Oncological treatment**	
No treatment	320 (65.7)
Adjuvant	103 (21.1)
Neoadjuvant	13 (2.7)
Palliative	51 (10.5)
**Route of administration**	
No treatment	320 (65.7)
Intravenous	61 (12.5)
Oral	96 (19.8)
Others (subcutaneous, intramuscular)	10 (2.1)
**Drug type**	
No treatment	320 (65.7)
Targeted therapy	25 (5.1)
Hormonotherapy	80 (16.4)
Immunotherapy	24 (4.9)
Chemotherapy	38 (7.8)

## Data Availability

Data available on request from the corresponding author. The data are not public ally available due to privacy or ethical restrictions.

## Supplementary Materials

The following supporting information can be downloaded at: https://www.mdpi.com/article/10.3390/curroncol29110675/s1, Table S1: Patient survey.

## Abbreviations

DK/NO: Do not know/no opinion.
