# Supplementary material for: Teleoncology: A Solution for Everyone? A Single-Center Experience with Telemedicine during the Coronavirus Disease 2019 (COVID-19) Pandemic
Source: Curr Oncol. 2022 Nov 11;29(11):8565–78. doi: 10.3390/curroncol29110675 (PMC9689494; doi:10.3390/curroncol29110675)
Supplement: Supplementary file 1 [file curroncol-29-00675-s001.zip › curroncol-1945648-supplementary.pdf]

Supplementary Materials

Patient survey

1- Who answered the survey?

- The patient
- A relative older than the patient
- A relative younger than the patient
- Others (care provider, friend, neighbour…)

2- Did the oncologists telephone visit comfort you regarding your disease and its control?

- Very
- Normal
- A little bit
- Not at all
- Do not know/no opinion

3- Did the oncologist telephone visit serve to resolve your doubts?

- Yes
- No
- Some of them, but not all.
- Do not know/no opinion

4- In the future, would you accept to switch some in-person to virtual visits?

- Yes
- No
- Do not know/no opinion

5- Do you think telemedicine may play a role in the future?

- Yes
- No
- Do not know/no opinion

6- Would you agree to be informed via telemedicine of imaging test results?

- Yes
- No
- Do not know/no opinion

7- Would you agree to be informed via telemedicine of laboratory test results?

- Yes
- No
- Do not know/no opinion

8- Would you agree to be informed via telemedicine of an intravenous treatment that you have to start receiving?

- Yes
- No
- Do not know/no opinion

9- Would you agree to be informed via telemedicine of an oral treatment that you have to start receiving?

- Yes
- No
- Do not know/no opinion

10- To what extent do you think that new technologies could facilitate the management of your disease?

- A lot
- Normal
- A little bit
- Any
- Do not know/no opinion

11- What is your knowledge of new technologies?

- Very high
- High
- Medium
- Low
- Null

12- Which tool would you like to use to contact your oncologist?

- Mobile application
- Video call
- Telephone call
- Text message
- E-mail
- Multiple answer (telephone, videocall, mobile APP, email…)
